# Revealing age-related changes in the intraocular microenvironment and senescence modulators using aqueous humor proteomics and machine learning

**DOI:** 10.3389/fcell.2025.1583330

**Published:** 2025-07-16

**Authors:** Xiaosheng Huang, Tiansheng Chou, Xinhua Liu, Kun Zeng, Liangnan Sun, Zonghui Yan, Shaoyi Mei, Wenqun Xi, Zongyi Zhan, Yi Liu, Songguo Dong, Siqi Liu, Jun Zhao

**Affiliations:** ^1^ Shenzhen Eye Medical Center, Shenzhen Eye Hospital, Southern Medical University, Shenzhen, China; ^2^ Department of Proteomics, Beijing Genomics Institute (BGI)-Shenzhen, Shenzhen, China; ^3^ National Medical Metabolomics International Collaborative Research Center, Xiangya Hospital, Central South University, Changsha, China; ^4^ Department of Ophthalmology, Shenzhen People’s Hospital (The Second Clinical Medical College, Jinan University, The First Affiliated Hospital, Southern University of Science and Technology), Shenzhen, China

**Keywords:** proteomes, aqueous humor, aging protein, senescence modulator, machine learning

## Abstract

**Background:**

In conjunction with age, aqueous humor (AH) proteomics can affect the occurrence and development of age-related eye diseases, which are poorly understood.

**Objective:**

We characterized the proteomic changes in AH throughout the aging process to better understand the aging mechanisms of the intraocular environment.

**Methods:**

We analyzed the AH proteomes of 33 older and 19 younger individuals using liquid chromatography–tandem mass spectrometry, from which we clustered similar expression trajectories of AH proteomics using local regression analysis. Aging proteins (APs) and their functional enrichment were evaluated using various statistical and bioinformatics methods, while aging modulators were predicted using multiple machine-learning models.

**Results:**

AH proteomic expression patterns exhibited various types of linear and nonlinear changes across the age groups. A set of 179 proteins identified as significant APs were enriched in various eye processes, such as detoxification, eye development, negative regulation of hydrolase activity, and humoral immune response. According to AH proteomics, hallmarks of aging include oxidative damage, defective extracellular matrices, and loss of proteostasis. A total of 11 APs were considered senescence signatures for predicting AH age with strong predictive ability. Furthermore, 22 APs were classified as modulators that may affect the aging process in the eye.

**Conclusion:**

These findings establish a framework for age-related changes in the AH proteome and provide potential senescence biomarkers and therapeutic targets for age-related eye diseases.

## 1 Introduction

Aging is an inherent biological process that affects most living organisms and leads to a gradual decrease in physiological functions over time, making it a significant risk factor for many chronic diseases (Campisi et al., 2019). Numerous studies have identified 12 hallmarks of aging, based on blood, brain, and other tissue samples, including cellular senescence, disabled macroautophagy, deregulated nutrient sensing, altered intercellular communication, chronic inflammation, genomic instability, telomere attrition, epigenetic alterations, loss of proteostasis, mitochondrial dysfunction, stem cell exhaustion, and dysbiosis (López-Otín et al., 2023). Age-related ocular diseases, such as age-related cataracts (ARCs), age-related macular degeneration, glaucoma, diabetic retinopathy, and retinal vein occlusion, are the result of numerous physiological and pathological processes that occur in the eye due to aging (Grossniklaus et al., 2013).

Proteomic studies of aqueous humor (AH) have greatly contributed to the understanding of the pathological changes in common eye diseases, including high myopia, cataracts, age-related macular degeneration, glaucoma, and diabetic retinopathy (Ji et al., 2015; Liu et al., 2021; Rinsky et al., 2021; Chiang et al., 2012). However, the majority of these studies did not include young individuals as healthy controls, thereby neglecting the effects of aging on AH and potentially introducing bias as little is known about the molecular changes that occur in the AH with age. To overcome these limitations, we compared the proteomics of AH between individuals across a wide range of ages to provide insights into the mechanisms of aging in the intraocular microenvironment. The application of artificial intelligence in chronic ocular diseases mainly includes the following aspects: disease diagnosis, clinical research, technical evaluation, and standardization construction (Xu and Yang, 2023; Yang et al., 2023; Ren et al., 2023). Proteomics supplies abundant biological data for machine learning (ML), which, in turn, enables powerful data analysis for proteomics. ML has been widely used to predict chronological age, protection, and risk factors in the plasma proteome to identify potential disease markers and effective anti-aging treatments for age-related diseases (Oh et al., 2023). This reciprocal relationship has been particularly transformative in ophthalmology. However, few studies have investigated whether the AH proteome contributes to the protective or deteriorating effects that occur during intraocular aging.

In this study, we compared the characteristics of the AH proteome among individuals across a wide range of ages (19–91 years) using liquid chromatography–tandem mass spectrometry in data-independent acquisition (DIA) mode. The DIA mode enables robust, high-coverage protein quantification from trace clinical specimens (e.g., single cells or microliter biofluids) through systematic MS/MS fragmentation and computational library matching, overcoming sensitivity limitations of traditional data-dependent approaches. Functionally annotated aging proteins (APs) were enriched in processes involving oxidative damage, defective extracellular matrix (ECM) organization, and loss of proteostasis. Furthermore, AH senescence signatures and proteome aging modulators were identified using multiple ML models. The results of this study not only revealed the common aging proteome patterns of AH but also enhanced our understanding of potential aging mechanisms within the intraocular microenvironment.

## 2 Materials and methods

### 2.1 Participants

A total of 86 participants, comprising 48 cortical cataract patients (age ≥50 years) and 38 young mild-to-moderate myopia donors (age <50 years; −6.0D <refractive error< −3.0D), were recruited from the Shenzhen Eye Hospital, China, from September 2021 to September 2023. All the subjects underwent a thorough ophthalmic evaluation, fundus examination, best-corrected visual acuity (BCVA) testing, intra-ocular pressure measurement, axial length (AL) assessment, corneal endothelial cell count, and optical coherence tomography. The inclusion criteria for the patient with cataracts were as follows: (1) age ≥50 years, (2) BCVA <0.3 LogMAR, and (3) moderate-to-severe lens opacity. The inclusion criteria for myopic patients with the clear lens setting, designated as the young group, were as follows: (1) age <50 years, (2) mild-to-moderate myopia and BCVA ≥0 LogMAR, and (3) clear lens. Exclusion criteria were (1) oculopathies, except for cataract and ametropia, such as diabetes cataract, keratitis, glaucoma, uveitis, and pseudoexfoliation syndrome; (2) any ocular traumas or ocular procedures; and (3) systemic diseases, such as hypercapnia, hyperuricemia, hyperthyroidism, and rheumatic disease. Finally, 33 ARC patients were set as the older group, and 19 mild-to-moderate myopia donors were set as the young group. All procedures performed in studies involving human participants were followed the ethical standards of the local Ethics Committee of Shenzhen Eye Hospital and the 1964 Helsinki Declaration and its later amendments or comparable ethical standards. Written informed consent was obtained from all study participants (reference code: SYLS 20200618-11 and date of approval: 28 June 2020). The clinical trial was registered at www.chictr.org as # ChiCTR2100042651.

### 2.2 Collection of the AH sample

AH samples were obtained from ARC patients undergoing cataract surgery and myopic patients with clear lenses undergoing posterior chamber phakic intraocular lens implantation surgery. A volume of 100–150 μL of AH was collected. Immediately following collection, AH samples were aliquoted into pre-chilled 1.5-mL LoBind microcentrifuge tubes (Eppendorf, Cat# 22431081). A 100 μL aliquot from each sample was used for protein extraction.

### 2.3 Extraction of the peptides derived from AH

AH protein extraction was performed as previously described with modifications (Lin et al., 2019). In brief, AH samples were homogenized in ice-cold lysis buffer containing 2% (w/v) RapiGest SF surfactant (Waters Corporation), 100 mM ammonium bicarbonate (NH_4_HCO_3_), 2 mM ethylenediaminetetraacetic acid, and 1 mM phenylmethylsulfonyl fluoride (pH 8.0). The suspension was subjected to pulsed ultrasonication (50 Hz, 3 s on/5 s off cycles) for 2 min at 4°C using a VCX130 probe sonicator (Sonics & Materials Inc., Newtown, CT, United States), followed by centrifugation at 20,000 × g for 10 min at 4°C. The supernatant was collected for protein quantification using the Bradford assay (Cat# P0006, Beyotime Biotechnology Co., Ltd.), with bovine serum albumin as the standard. Aliquots containing 10 μg of protein were reduced with 10 mM dithiothreitol at 56°C for 30 min and alkylated with 55 mM iodoacetamide in the dark at 25°C for 45 min. Trypsin digestion was performed at a 1:50 (w/w) trypsin-to-protein ratio in 100 mM NH_4_HCO_3_ at 37°C for 16 h. Resulting peptides were desalted using stage-tip columns packed with OLIGO R3 Reversed-Phase Resin (30 μm, nest group) and vacuum-dried for LC-MS/MS analysis.

### 2.4 Quantitative proteomics using DIA

The dried AH peptides were assessed by LC-MS/MS using an Ultimate 3000 Nano-LC System coupled to the Orbitrap Fusion Lumos Mass Spectrometer (Thermo Fisher Scientific, United States) and operated in DIA mode with a duration of 120 min. The parameters were referenced from our laboratory routine sets (Fan et al., 2021). To generate the spectrum library for protein identification, samples were reconstituted with mobile phase A (2% ACN and 0.1% FA) and centrifuged at 20,000 × g for 10 min, and the supernatant was collected for injection. Separation was carried out using the UltiMate 3000 UHPLC System. The sample was first enriched in the trap column and desalted, then introduced into a tandem self-packed C18 column (150 μm internal diameter, 1.8 μm column size, and 35 cm column length), and was separated at a flow rate of 500 nL/min using the following effective gradient: 0–5 min, held at 5% mobile phase B (98% ACN, 0.1% FA); 5–130 min, mobile phase B linearly increased from 5% to 25%; 130–150 min, mobile phase B increased from 25% to 35%; 150–160 min, mobile phase B increased from 35% to 80%; 160–175 min, held at 80% mobile phase B; 175–175.5 min, mobile phase B decreased from 80% to 5%; and 175.5–180 min, held at 5% mobile phase B. The nanoliter liquid-phase separation outlet was directly connected to the mass spectrometer. To generate the spectrum library, the MS/MS signals were acquired in data-dependent acquisition mode with the following parameter settings: full-scan MS spectra (350–1,500 m/z) with a resolution of 120,000, high-energy collision dissociation using 28% relative energy, and MS/MS scan at 15,000 resolutions (Yan et al., 2022).

### 2.5 Peptide analysis based on MS/MS signals and data processing

The MS/MS signals elicited from the DDA mode were input to a search engine, MaxQuant (v.1.6.0.1), and were searched against human SWISS-PROT entries from the UniProtKB database (UniProt, release 2018_02), with the following parameters: maximum missed cleavage at 1, fixed modification at cysteine carbamidomethylation, variable modification at methionine oxidation and N-terminal protein acetylation, and minimal peptide length at seven amino acids. The maximum false discovery rate (*FDR*) for peptide or protein identification was set at 0.01. The MaxQuant output was used to generate the spectral library for DIA analysis. The MS/MS signals acquired in DIA mode were input into Spectronaut (v.12.4, BIOGNOSYS, Switzerland) for peptide and protein quantification using RT calibration by iRT. Based on the target-decoy model, the false positive rate of peptides and proteins was set to less than 0.01, thereby ensuring significant quantitative results.

After overlaying duplicates and removing the missing match peptide, missing values were imputed using the generalized mass spectrum method, following an assessment of 23 commonly used missing value imputation methods by NAguideR (Wang et al., 2020) (https://www.omicsolution.com/wukong/NAguideR/#, Supplementary Table S1). Protein abundances were log2-transformed.

### 2.6 Principal component analysis, cluster analysis of the AH proteome, and functional enrichment of APs

The partial least squares-discriminant analysis was implemented using the Wukong data analysis platform (https://www.omicsolution.org/). To cluster the AH proteome based on similar expression trajectories across aging, a local regression analysis (LOESS function) from the R stats package (v4.2.2) with a span of 0.75 was used, as described by Coenen et al. (2023). The *Z*-scores were computed for each protein individually. To reduce noise and variability, the following LOESS model was applied separately to each protein: protein expression ∼ age. The hclust function of the R stats package was applied for unsupervised hierarchical clustering analysis. The hypergeometric test was used to assess the enrichments of APs in the defined clusters using the phyper function in R. The KEGG, GO, and Reactome databases with the R packages clusterProfiler (v4.6.2) and ReactomePA (v1.42.0) were used to identify the biological relevance of APs. HumanBase functional protein module analysis was used for identifying cohesive gene clusters and representing the eye-special process of APs (Greene et al., 2015) (https://hb.flatironinstitute.org/gene/).

### 2.7 Age prediction, Δage estimation, identification of accelerated, decelerated, and chronological agers, and factors of the AH proteome

The detailed methods of ML models were described by Coenen et al. (2023) and Oh et al. (2023). In brief, 33 individuals were randomly selected as the training set, and the remaining individuals (*n* = 19) were selected as the testing set. Next, the least absolute shrinkage and selection operator (LASSO) models (alpha = 1, minimum lambda value as estimated after 10-fold cross validation) using all APs as input variables were fitted to determine whether the AP can predict the chronological age. The LASSO model was repeated 1,000 times, and the frequency of the variable being retained in the models was more than 500 times across all models to identify key APs in the training set. Next, the ridge regression model (alpha = 0) was used to obtain age predictions using only the reduced APs described above and gender as the input in the training set and was validated in the testing set. A novel linear model was used to estimate the chronological age, which improved upon the fitted ridge model (linear-model LM-predicted age ∼ chronological age), as shown in the following equation: unbiased age prediction = (predicted age − intercept of LM)/coefficient of LM. This unbiased estimate of the predicted age was used to subtract the chronological age to obtain our unbiased Δage. In addition, the mean average error (MAE) was calculated for each dataset individually as follows: MAE = Σ(| Δage|)/number of Δage estimates. Finally, the linear models for each AP were fitted to identify which APs were significantly associated with Δage: protein expression ∼ age + gender + BMI+ Δage. The biological age group was treated as a category, with decelerated agers (DAs) defined as Δage ≤ −5 years, accelerated agers (AAs) as Δage of ≥5 years, and chronological agers (CAs) as |ΔAge| < 2 years. To identify which APs were significantly associated with the biological age group, the following linear model was fitted for each AP: protein expression ∼ age + gender + BMI+ biological age group.

### 2.8 Statistical analysis

Independent-sample *t*-tests were used to compare age and AL between the two groups. The Wilcoxon rank-sum test was performed to evaluate the significance of proteins between the two groups. Linear regression modeling was used to test the effect of age on protein expression levels while correcting for most of the available metadata to correct for possible confounding effects: protein expression ∼ age + gender + BMI. The APs were the preserved set of DEPs satisfied with significant proteins associated with age in the linear regression modeling test. *P*-values were adjusted using the FDR (*Padj*), and *P* value and *Padj* < 0.05 were considered statistically significant.

## 3 Results

### 3.1 Demographic data

This study included 33 older patients with ARC (33 eyes) and 19 younger patients as healthy controls (19 eyes). The mean age of the patients in the older group (65.55 ± 11.30 years) was significantly greater than that of the younger group (26.95 ± 5.13 years; *P* < 0.001; Table 1). No significant difference was observed in the mean AL between the older and younger groups (24.54 ± 2.39 mm vs. 25.23 ± 0.59 mm; *P* > 0.05; Table 1). The lens opacities of the patients with cataracts were classified according to Lens Opacities Classification System III. The clinical features of all subjects are listed in Supplementary Table S2.

**TABLE 1:** Demographic and clinical features of the older and young groups.

Basic characteristics	Older group (*n* = 33)	Young group (*n* = 19)	*P*
Age (years ± SD)	65.55 ± 11.30	26.95 ± 5.13	< 0.05
AL (mm ± SD)	24.54 ± 2.39	25.23 ± 0.59	0.221
Lens transparency	Opacified lens	Clear lens	

P-value from independent-sample *t*-tests for difference in age and axial length between the older and control groups.

n, number of subjects; *P*, *P*-value; AL, axial length.

### 3.2 Principal component analysis and expression trajectory analysis of the AH proteome during aging

**FIGURE 1:**
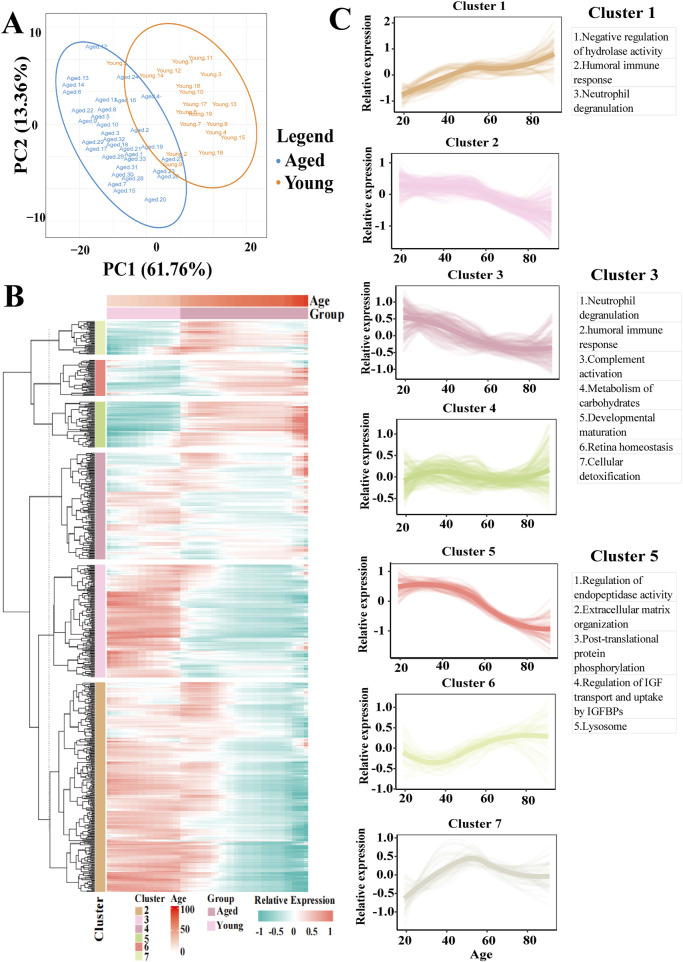
Proteomic overview of the AH and clustering analysis of the complete AH proteome based on expression trajectories across age. **(A)** Principal component analysis plots for the proteomes responsive to young and older AH. **(B)** Heatmap of identified clusters following unsupervised analyses of all AH proteins based on expression trajectories across age. Green color indicates a relative decrease, whereas red color indicates a relative increase compared to the mean expression over time. **(C)** Expression trajectories of the seven clusters. Clusters 1, 3 and 5 exhibited linear up- or downregulation and retained based on a hypergeometric test (*Padj* < 0.05). The major processes associated with clusters 1, 3 and 5 are listed.

We initially utilized partial least-squares discriminant analysis to assess the distinction between the two groups of participants, thereby clearly identifying the older and younger groups (Figure 1A). Following peptide-spectrum matching, stringent protein inference (FDR <1%), and rigorous missing-value imputation processing, our analysis yielded 634 high-confidence proteins meeting the identification criteria of having ≥2 unique peptides per protein group (Supplementary Table S3). The AH proteome was clustered based on trajectories with similar expression across age groups to determine which changes occur in the aging intraocular environment (Figure 1B). Seven clusters with comparable expression trajectories were identified, reflecting diverse patterns of expression changes during the aging process, including both linear and nonlinear up- or downregulations, indicating varying degrees of involvement in these processes (Supplementary Table S4).

Clusters 1, 3, and 5 exhibited hypergeometric distributions (*P*_*adj*_ < 0.05). Analyses of functional enrichment across the GO, KEGG, and Reactome databases revealed numerous enrichments in terms of functions, cellular components, and pathways within these clusters (Supplementary Table S5) as follows: Cluster 1—regulation of peptidase activity, humoral immune response, and neutrophil degranulation; Cluster 3—neutrophil degranulation, humoral immune response, complement activation, carbohydrate metabolism, developmental maturation, retinal homeostasis, and cellular detoxification; and Cluster 5—regulation of peptidase activity, ECM organization, neutrophil degranulation, and other processes.

### 3.3 Identification of APs in the AH, functional enrichment analysis of APs, and association between APs and the degree of lens opacity

**FIGURE 2:**
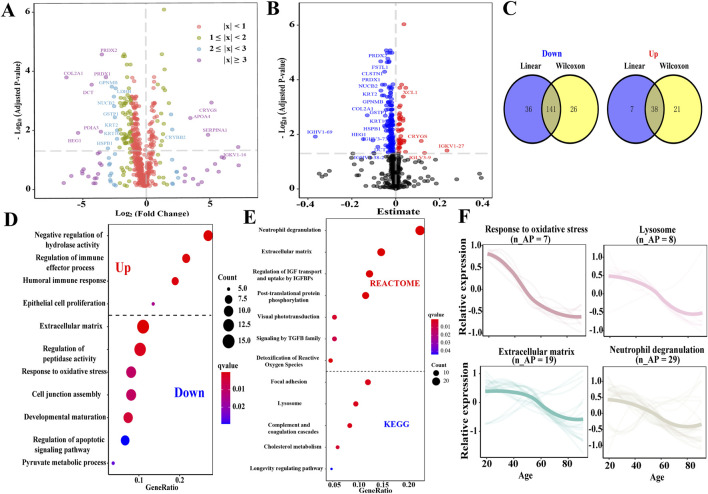
Identification of APs in AH and enrichment function analysis of APs. **(A)** Volcano plot depicts the DEPs of AH identified by the Wilcoxon rank-sum test. The top 20 DEPs were depicted (*Padj* < 0.05). Purple dot, |log_2_foldchange| ≥ 3; blue dot, 2 ≤ |log_2_foldchange| ≤ 3; green dot, 1 ≤ |log_2_foldchange| ≤ 2; and red dot, |log_2_foldchange| ≤ 1. **(B)** Volcano plot depicts the relationship in the AH proteome across age using a comparable linear regression model approach. The red plot represents a positive correlation between the protein and age, while the blue plot represents a negative correlation with age. **(C)** The Venn diagram depicts the APs of AH. **(D)** Overview displaying a wide variety of significant Gene Ontology functional enrichments of the subsets according to upregulated and downregulated APs. Dot size represents the number of proteins involved, and dot color represents significance levels as q-value. **(E)** Overview displaying a wide variety of significant pathways of APs through the KEGG and Reactome enrichment analyses. **(F)** Expression trajectories of the significant functional APs. n_APs, the matched number of aging pathway components in APs.

We conducted a proteomic analysis of the composition of AH in older and younger individuals to identify potential mechanisms underlying AH senescence and impaired lens transparency. There were 226 differentially expressed proteins (DEPs) in the older group compared with the younger group, comprising 59 upregulated and 167 downregulated DEPs (all *P*_*adj*_ < 0.05; Figure 2A). Next, a comparable linear regression model was used to assess 222 unique proteins that were significantly associated with age (all *P*_*adj*_ < 0.05; Figure 2B), of which 45 were positively related to age, while 167 were negatively correlated. Finally, 141 downregulated and 38 upregulated proteins were identified as APs in the AH, which satisfied the Wilcoxon rank-sum test and comparable linear regression model approach (Figure 2C; Supplementary Table S6). The top 10 up- and downregulated APs are listed in Table 2. A total of 84 APs were detected in matched plasma samples (unpublished data).

**TABLE 2:** Top 10 upregulated and downregulated APs in AH across age.

Upregulated	Protein	Abbreviation	Mean expression		Log_2_FC	*P* ^*^
			Older group	Young group		
1	Gamma-crystallin S	CRYGS	32.28	26.98	5.30	< 0.05
2	Lipocalin 2	LCN2	14.82	12.88	1.95	< 0.05
3	C16orf46	-	14.14	12.50	1.64	< 0.05
4	Apolipoprotein A2	APOA2	20.54	18.92	1.62	< 0.05
5	Serine protease inhibitor Kazal-type 1	SPINK1	12.74	11.15	1.59	< 0.05
6	X-C motif chemokine ligand 1	XCL1	16.78	15.24	1.54	< 0.05
7	Left-right determination factor 2	LEFTY2	12.54	11.02	1.51	< 0.05
8	Transmembrane protein 198	TMEM198	17.20	15.80	1.41	< 0.05
9	Serum amyloid A-4	SAA4	15.23	13.84	1.39	< 0.05
10	THAP domain containing 4	THAP4	16.77	15.44	1.34	< 0.05
Downregulated						
1	Immunoglobulin heavy variable 1–69	IGHV1-69	41.77	57.56	−15.80	< 0.05
2	Collagen type II alpha 1 chain	COL2A1	20.34	26.46	−6.12	< 0.05
3	Heart development protein with EGF like domains 1	HEG1	16.02	21.23	−5.22	< 0.05
4	Dopachrome tautomerase	DCT	10.17	14.30	−4.13	< 0.05
5	Protein disulfide isomerase family A member 3	PDIA3	18.91	22.33	−3.42	< 0.05
6	Peroxiredoxin 2	PRDX2	13.36	16.68	−3.32	< 0.05
7	Peroxiredoxin 1	PRDX1	10.70	13.71	−3.01	< 0.05
8	Glycoprotein nmb	GPNMB	12.16	14.70	−2.54	< 0.05
9	Nucleobindin 2	NUCB2	12.26	14.64	−2.38	< 0.05
10	Glutathione S-transferase pi 1	GSTP1	13.12	15.48	−2.37	< 0.05

P^*^, The *P*-value was corrected using the FDR.

Various processes and pathways associated with the APs were enriched to understand their biological processes (Supplementary Table S7). The upregulated APs were enriched in the negative regulation of hydrolase activity, regulation of immune effector processes, humoral immune response, and epithelial cell proliferation. In contrast, the downregulated APs were enriched in the ECM organization, regulation of peptidase activity, response to oxidative stress, regulation of the apoptotic signaling pathway, and pyruvate metabolic process (Figure 2D). The pathways enriched in the KEGG and Reactome databases included neutrophil degranulation, regulation of insulin-like growth factor (IGF) transport and uptake by insulin-like growth factor-binding protein (IGFBP), post-translational protein phosphorylation (PTM), visual phototransduction (VP), signaling by transforming growth factor (TGF-β) family members, detoxification of reactive oxygen species, complement and coagulation cascades, cholesterol metabolism, and longevity-regulating pathways (Figure 2E). Several processes demonstrated a linear downregulation pattern, such as response to oxidative stress, lysosomes, ECM organization, and neutrophil degranulation (Figure 2F).

The primary biological characteristic of older individuals in this study was lens opacity, with strong correlations between the degree of lens opacity and expression levels of all up- and downregulated APs (*r* = 0.68 and −0.73, respectively; *P* < 0.05; Supplementary Table S8). The degree of lens opacity was strongly correlated with 12 downregulated APs and 1 upregulated AP (0.60 < |r| < 0.80; *P*_*adj*_ < 0.05). The remaining APs were only mildly or moderately correlated with the degree of lens opacity (Supplementary Table S8).

### 3.4 Identification of the differences in APs and age-related pathways between AH and plasma and module analysis of the relationships between APs and eye processes

**FIGURE 3:**
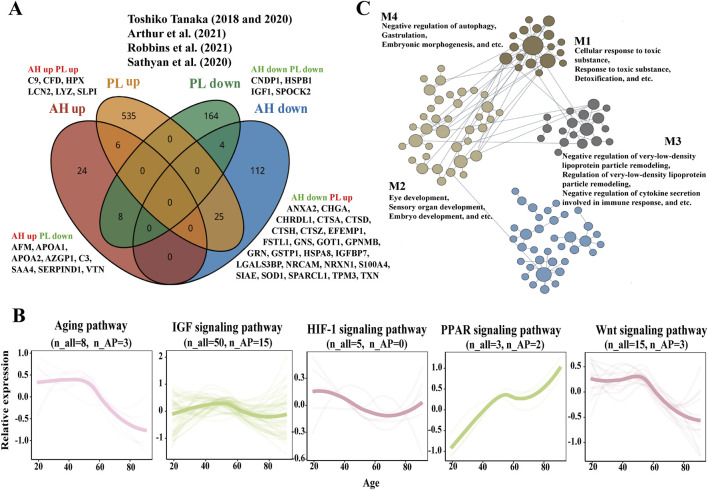
Comparison of APs and aging related pathways in AH and plasma and process-specific functional analysis of APs on eye. **(A)** The Venn diagram depicts the results of the integration across the AH APs of our study and plasma APs from previous studies (Tanaka et al., 2020; Tanaka et al., 2018; Moaddel et al., 2021; Sathyan et al., 2020; Arthur et al., 2021). **(B)** Expression trajectories of the aging-related pathways in AH. **(C)** The network of APs contributed to the eye-specific processes in four modules. n_all, the matched number of aging pathway components in all AH proteomics. n_APs, the matched number of aging pathway components in APs.

A total of 568 upregulated and 177 downregulated plasma APs were identified in previous studies (Tanaka et al., 2018; Tanaka et al., 2020; Moaddel et al., 2021; Sathyan et al., 2020; Arthur et al., 2021). Only 10 APs exhibited consistent expression trends in both the AH and plasma, whereas the expression of 33 APs showed the opposite trend (Figure 3A; Supplementary Table S9).

Proteomic studies of various materials (plasma, serum, urine, saliva, and other tissues) have revealed various pathways related to biological aging, including IGF, hypoxia-inducible factor-1 (HIF-1), and cytokine signaling metabolic pathways (Moaddel et al., 2021). AH proteomics might provide some insights into age-related signaling pathways, such as aging, IGF, HIF-1, peroxisome proliferator-activated receptor (PPAR), and Wnt signaling pathways in the intraocular environment (Figure 3B). The expression of genes related to aging and the Wnt signaling pathway decreased with age, whereas the expression of genes related to the PPAR signaling pathway increased with age. The expression trajectories of IGF and the HIF-1 signaling pathway seemed to be stable.

Using HumanBase functional module analysis, we identified cohesive gene clusters and process-specific functional relationship networks involving APs that contribute to eye processes (Greene et al., 2015). The APs were divided into four modules: M1—cellular response to toxic substances, response to toxic substances, and detoxification; M2—eye development, sensory organ development, and embryo development; M3—negative regulation of very-low-density lipoprotein particle remodeling, regulation of very-low-density lipoprotein particle remodeling, and negative regulation of cytokine secretion involved in the immune response; and M4—negative regulation of autophagy, gastrulation, and embryonic morphogenesis (Figure 3C; Supplementary Table S10).

### 3.5 ML models predict the age of AH and identify the aging modulator in AH

**FIGURE 4:**
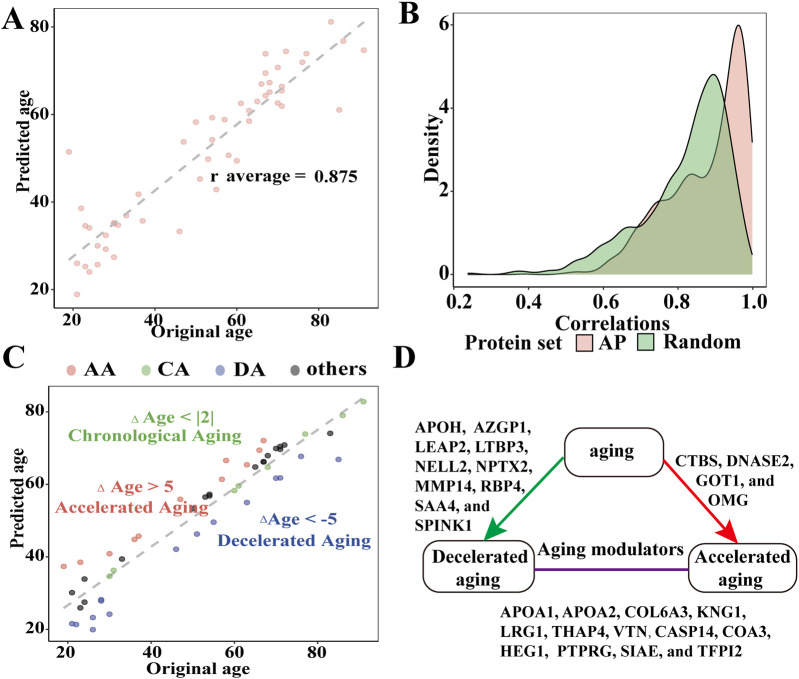
Evaluating the efficacy of APs to predict AH age and identify the components affecting aging processes in AH. **(A)** Relationship between the predicted and chronological age across 1,000 models (r average = 0.875). **(B)** Distributions of the 1,000 correlations between the original and predicted ages obtained from models using all 179 APs and 179 random proteins of AH. **(C)** Identification of chronological agers (CAs, green), decelerated agers (DAs, red), and accelerated agers (AA, blue) based on the average Δage estimates using 179 APs. **(D)** Graphical summary of the aging modulators in AH (all *P* < 0.05).

On average, we found strong correlations between the original and predicted ages across all 1,000 models (rAP_age_ = 0.875) based on the following 11 APs: AZGP1, COL1A2, COL6A3, CRYGS, CTBS, GSTO1, LEFTY2, LINGO1, LYZ, NPTX2, and RBP4 (> 500 LASSO regression analyses were selected; Figure 4A). The AUCs of these APs were more than 0.724, and the receiver operating characteristic (ROC) curves of these APs are presented in Supplementary Figure S2.

We also tested models with sets of random proteins of similar size from the AH to determine whether models consisting solely of APs were superior predictors of age compared to other proteins in the AH. Consequently, models for the 11 APs outperformed the model that included 179 random AH proteins (Figure 4B). To address collinearity, we repeated the age prediction analyses using ridge regression. This reduced subset of APs demonstrated strong predictive validity across 1,000 models (average r = 0.92).

Next, we calculated the average predicted age using the expression levels of the 179 random APs. We compared the average predicted age with the chronological age of each individual to estimate Δage while statistically controlling for chronological age, sex, and body mass index. We observed that the ranges of the Δage values followed an approximately normal distribution (Shapiro–Wilk test, *P* = 0.38; Figure 4C). The mean Δage was 0 with a mean average error of 5.537 years. A total of 22 APs with significant correlations with age were identified as potential aging modulators in the AH (all *P* < 0.05). Among these aging modulators, 11 were positively correlated with age (APOA1, APOA2, APOH, COL6A3, KNG1, LEAP2, LRG1, SAA4, SPINK1, THAP4, and VTN), while the remaining modulators (CASP14, COA3, CTBS, GOT1, HEG1, NELL2, NPTX2, OMG, PTPRG, SIAE, and TFPI2) were negatively correlated (Figure 4D; Supplementary Table S11). We compared AA and DA with CA to identify the proteomic data associated with decelerated aging. We identified ten proteins (LTBP3, NELL2, NPTX2, APOH, AZGP1, LEAP2, MMP14, RBP4, SAA4, and SPINK1) that might decelerate interocular aging, whereas CTBS, DNASE2, GOT1, and OMG might accelerate interocular aging (all *P* < 0.05) (Supplementary Table S12).

## 4 Discussion

Age-related eye diseases, including ARC, glaucoma, diabetic retinopathy, and age-related macular degeneration, frequently occur as part of the aging process and are becoming more prevalent in older populations (Grossniklaus et al., 2013). AH is regarded as a special body fluid, and AH proteins may diffuse from the ciliary body stroma of plasma-derived proteins and be expressed in the intraocular tissue. Alterations in the proteome of the AH play a significant role in various pathological processes within the intraocular environment, offering valuable insights into the pathophysiology of age-related eye diseases (Ji et al., 2015; Liu et al., 2021; Rinsky et al., 2021; Chiang et al., 2012). In this study, we classified seven distinct expression trajectories across the different age groups. Three of these clusters corresponded to a hypergeometric distribution and exhibited a linear expression trajectory, which may provide a strategy for slowing the aging process in intraocular tissues by focusing on neutrophil degranulation, humoral immune response, cellular detoxification, and carbohydrate metabolism. Several age-related pathways previously associated with biological aging in the plasma were found to be involved in aging processes within the eye, including aging, IGF, HIF-1, PPAR, and Wnt signaling pathways (Moaddel et al., 2021). The composition and expression levels of proteins in AH differ from those in the plasma because of the blood–aqueous barrier (Freddo, 2013). We found that only 10 APs exhibited consistent expression trends in both the AH and plasma, while 33 APs showed contrasting expression trends (Figure 3A) (Tanaka et al., 2018; Tanaka et al., 2020; Moaddel et al., 2021; Sathyan et al., 2020; Arthur et al., 2021). Therefore, it is inaccurate to assume that APs in the plasma are functionally equivalent to those in the AH as APs in the AH may play roles in eye-specific processes, such as detoxification, eye development, negative regulation of very-low-density lipoprotein particle remodeling, and negative regulation of autophagy (Figure 3B). According to the CellAge database, 13 APs, namely, ABI3BP, CTSD, GRN, HSPB1, HYOU1, NOTCH1, NRSN2, SOD1, TGFB2, TPP1, TXN, WIF1, and LCN2, have been proven to exacerbate deterioration due to aging in several functional experimental cell lines (Tacutu et al., 2018).

The AH contains a wide variety of APs in low quantities, which contribute to the aging process in the eyes through three aging-related mechanisms, namely, oxidative damage, defective ECM organization, and loss of proteostasis. The AH serves as a significant source of antioxidants that directly supplement adjacent tissues such as the lens, corneal endothelial cells, and the trabecular meshwork (Liu et al., 2021; Hsueh et al., 2022). We found that the antioxidant system in AH exhibited a significant imbalance in older individuals, with multiple antioxidant factors, including GSTO1, GSTP1, PRDX1, PRDX2, PRDX6, SOD1, and TXN, simultaneously decreasing. The reduced expression of these antioxidant factors may impede the efficient removal of active oxygen compounds, such as hydrogen peroxide and superoxide, potentially worsening the cellular damage produced by free radicals. This imbalance has the potential to disrupt the cellular redox balance, hindering the detoxification of oxidative stress substances, which, consequently, may compromise the functionality and structural integrity of proteins within the cells, thereby obstructing the effective conversion and elimination of harmful substances inside the cells. The antioxidant activity of AH in older individuals noticeably decreases with physiological aging; therefore, the restoration of these antioxidant factors may serve as an anti-aging therapy for age-related diseases.

The ECM organization provides a microenvironment for various cell types, imparts cellular structural and mechanical support, and regulates cellular homeostasis and signaling (Selman and Pardo, 2021; López-Otín et al., 2023). Therefore, changes in the ECM organization affect the permeability of nutrients and metabolites to the lens, decrease corneal biomechanical stability, alter the corneal shape, and impede the outflow of AH (Pouw et al., 2021; Wederell and de Iongh, 2006). We found that 19 types of ECM organization were significantly altered in the AH of older individuals, resulting in the disruption of multiple aspects of intraocular cell homeostasis and normal functioning. Additionally, the decrease in proteostasis due to age involves reduced translation, impaired unfolded protein response, endoplasmic reticulum stress response, compromised chaperone function, and impaired function of the ubiquitin–proteasome system and autophagy–lysosome pathway (Weinberg et al., 2022). In the AH of older individuals, hydrolase activity is disrupted and lysosomal constituents decrease, which may be involved in the pathological mechanisms of lens development and homeostasis, glaucoma, and age-related macular degeneration (Weinberg et al., 2022). The molecular chaperones HSPA6, HSPA8, and HSPB1, whose expression decreased in the AH of older individuals, impeded the ability of eye cells to respond to stress as they influence protein quality control in the intraocular tissues under stress (Weinberg et al., 2022; Fu et al., 2023). Antioxidation, ECM organization repair, and maintenance of protein homeostasis emerge as key therapeutic directions for age-related eye diseases. With the ongoing advancements in artificial intelligence, ML plays a role in predicting disease markers and therapeutic targets after analyzing omics data (Oh et al., 2023). To investigate the APs involved in the aging process, we established a model using LASSO regression, which was subsequently validated using ridge regression. A group of 11 APs (AZGP1, COL1A2, COL6A3, CRYGS, CTBS, GSTO1, LEFTY2, LINGO1, LYZ, NPTX2, and RBP4) were identified as senescence biomarkers of AH, possessing a stronger predictive ability than random AH proteins. The early risk stratification of age-related eye diseases can be achieved through the detection of these senescence biomarkers in AH. Among the aforementioned senescence biomarkers, COL1A2, COL6A3, CRYGS, LINGO1, LYZ, NPTX2, and RBP4 are known to be associated with age-related diseases such as ARC, dermal aging, and Alzheimer’s and Parkinson’s diseases (Hooi et al., 2012; Quan et al., 2021; Savić et al., 2023; de Laat et al., 2015; Sassi et al., 2016; Xiao et al., 2017; Goodman, 2006). Additionally, 22 APs were identified as potential modulators of aging in the AH, indicating their potential contribution to maintaining the balance of intraocular aging across different age groups. These 22 modulators represent promising therapeutic targets for retarding intraocular aging and need further intensive investigation to confirm.

This study had some limitations. First, the sample size in our study was insufficient due to the ethical limitations of using AH from healthy individuals. Second, there were no suitable AH proteome data to confirm our results at the time of our analysis. Third, the findings were not further validated using additional detection methods such as Western blot, ELISA, and MRM. Future research could incorporate these techniques to more rigorously confirm our observations and enhance the reliability of the conclusions drawn from our analysis. In summary, the proteomic analysis of AH provides an alternative approach to understanding the pathological mechanisms associated with aging. The results of this analysis provide novel insights into the senescence signature of AH, classify different senescence biomarkers in AH and plasma, and reveal new potential treatments for slowing the aging process of the eye using ML.

## Data Availability

The mass spectrometry proteomics data have been deposited to the ProteomeXchange Consortium (http://proteomecentral.proteomexchange.org) via the iProX partner repository with the dataset identifier PXD064856.

## References

[B1] Arthur L. Esaulova E. Mogilenko D. A. Tsurinov P. Burdess S. Laha A. (2021). Cellular and plasma proteomic determinants of covid-19 and non-covid-19 pulmonary diseases relative to healthy aging. Nat. Aging 1 (6), 535–549. 10.1038/s43587-021-00067-x 37117829

[B3] Campisi J. Kapahi P. Lithgow G. J. Melov S. Newman J. C. Verdin E. (2019). From discoveries in ageing research to therapeutics for healthy ageing. Nature 571 (7764), 183–192. 10.1038/s41586-019-1365-2 31292558 PMC7205183

[B4] Chiang S. Y. Tsai M. L. Wang C. Y. Chen A. Chou Y. C. Hsia C. W. (2012). Proteomic analysis and identification of aqueous humor proteins with a pathophysiological role in diabetic retinopathy. J. Proteomics 75 (10), 2950–2959. 10.1016/j.jprot.2011.12.006 22200677

[B5] Coenen L. Lehallier B. de Vries H. E. Middeldorp J. (2023). Markers of aging: unsupervised integrated analyses of the human plasma proteome. Front. Aging. 4, 1112109. 10.3389/fragi.2023.1112109 36911498 PMC9992741

[B6] de Laat R. Meabon J. S. Wiley J. C. Hudson M. P. Montine T. J. Bothwell M. (2015). Lingo-1 promotes lysosomal degradation of amyloid-Β protein precursor. Pathobiol. Aging Age Relat. Dis. 5, 25796. 10.3402/pba.v5.25796 25758563 PMC4355507

[B7] Fan Y. Bai B. Liang Y. Ren Y. Liu Y. Zhou F. (2021). Proteomic profiling of gastric signet ring cell carcinoma tissues reveals characteristic changes of the complement cascade pathway. Mol. Cell Proteomics 20, 100068. 10.1016/j.mcpro.2021.100068 33676000 PMC8121970

[B8] Freddo T. F. (2013). A contemporary concept of the blood-aqueous barrier. Prog. Retin Eye Res. 32, 181–195. 10.1016/j.preteyeres.2012.10.004 23128417 PMC3544162

[B2] Fu J. L. Zheng S. Y. Wang Y. Hu X. B. Xiao Y. Wang J. M. (2023). HSP90β prevents aging-related cataract formation through regulation of the charged multivesicular body protein (CHMP4B) and p53. Proc. Natl. Acad. Sci. U.S.A. 120 (31), e2221522120. 10.1073/pnas.2221522120 37487085 PMC10400967

[B9] Goodman A. B. (2006). Retinoid receptors, transporters, and metabolizers as therapeutic targets in late onset alzheimer disease. J. Cell Physiol. 209 (3), 598–603. 10.1002/jcp.20784 17001693

[B10] Greene C. S. Krishnan A. Wong A. K. Ricciotti E. Zelaya R. A. Himmelstein D. S. (2015). Understanding multicellular function and disease with human tissue-specific networks. Nat. Genet. 47 (6), 569–576. 10.1038/ng.3259 25915600 PMC4828725

[B11] Grossniklaus H. E. Nickerson J. M. Edelhauser H. F. Bergman L. A. Berglin L. (2013). Anatomic alterations in aging and age-related diseases of the eye. Invest Ophthalmol. Vis. Sci. 54 (14), 23–27. 10.1167/iovs.13-12711 PMC3864374 24335063

[B12] Hooi M. Y. Raftery M. J. Truscott R. J. (2012). Age-dependent deamidation of glutamine residues in human Γs crystallin: deamidation and unstructured regions. Protein Sci. 21 (7), 1074–1079. 10.1002/pro.2095 22593035 PMC3403444

[B13] Hsueh Y. J. Chen Y. N. Tsao Y. T. Cheng C. M. Wu W. C. Chen H. C. (2022). The pathomechanism, antioxidant biomarkers, and treatment of oxidative stress-related eye diseases. Int. J. Mol. Sci. 23 (3), 1255. 10.3390/ijms23031255 35163178 PMC8835903

[B14] Ji Y. Rong X. Ye H. Zhang K. Lu Y. (2015). Proteomic analysis of aqueous humor proteins associated with cataract development. Clin. Biochem. 48 (18), 1304–1309. 10.1016/j.clinbiochem.2015.08.006 26265347

[B37] Lin Z. Zhang Y. Pan H. Hao P. Li S. He Y. (2019). Alternative strategy to explore missing proteins with low molecular weight. J. Proteome. Res. 18 (12), 4180–4188. 10.1021/acs.jproteome.9b00353 31592669

[B15] Liu X. Liu X. Wang Y. Sun H. Guo Z. Tang X. (2021). Proteome characterization of glaucoma aqueous humor. Mol. Cell Proteomics 20, 100117. 10.1016/j.mcpro.2021.100117 34214668 PMC8367844

[B16] López-Otín C. Blasco M. A. Partridge L. Serrano M. Kroemer G. (2023). Hallmarks of aging: an expanding universe. Cell 186 (2), 243–278. 10.1016/j.cell.2022.11.001 36599349

[B17] Moaddel R. Ubaida-Mohien C. Tanaka T. Lyashkov A. Basisty N. Schilling B. (2021). Proteomics in aging research: a roadmap to clinical, translational research. Aging Cell 20 (4), e13325. 10.1111/acel.13325 33730416 PMC8045948

[B18] Oh H. S. Rutledge J. Nachun D. Pálovics R. Abiose O. Moran-Losada P. (2023). Organ aging signatures in the plasma proteome track health and disease. Nature 624 (7990), 164–172. 10.1038/s41586-023-06802-1 38057571 PMC10700136

[B19] Pouw A. E. Greiner M. A. Coussa R. G. Jiao C. Han I. C. Skeie J. M. (2021). Cell-matrix interactions in the eye: from cornea to choroid. Cells 10 (3), 687. 10.3390/cells10030687 33804633 PMC8003714

[B20] Quan T. Xiang Y. Liu Y. Qin Z. Yang Y. Bou-Gharios G. (2021). Dermal fibroblast Ccn1 expression in mice recapitulates human skin dermal aging. J. Invest Dermatol. 141 (4), 1007–1016. 10.1016/j.jid.2020.07.019 32800875 PMC7881053

[B21] Ren P. F. Tang X. Y. Yu C. Y. Zhu L. L. Yang W. H. Shen Y. (2023). Evaluation of a novel deep learning based screening system for pathologic myopia. Int. J. Ophthalmol. 16 (9), 1417–1423. 10.18240/ijo.2023.09.07 37724265 PMC10475629

[B22] Rinsky B. Beykin G. Grunin M. Amer R. Khateb S. Tiosano L. (2021). Analysis of the aqueous humor proteome in patients with age-related macular degeneration. Invest Ophthalmol. Vis. Sci. 62 (10), 18. 10.1167/iovs.62.10.18 PMC8374990 34406330

[B23] Sassi C. Ridge P. G. Nalls M. A. Gibbs R. Ding J. Lupton M. K. (2016). Influence of coding variability in app-Aβ metabolism genes in sporadic Alzheimer's disease. PLoS One 11 (6), e0150079. 10.1371/journal.pone.0150079 27249223 PMC4889076

[B24] Sathyan S. Ayers E. Gao T. Milman S. Barzilai N. Verghese J. (2020). Plasma proteomic profile of frailty. Aging Cell 19 (9), e13193. 10.1111/acel.13193 32762010 PMC7511877

[B25] Savić R. Yang J. Koplev S. An M. C. Patel P. L. O'Brien R. N. (2023). Integration of transcriptomes of senescent cell models with multi-tissue patient samples reveals reduced Col6a3 as an inducer of senescence. Cell Rep. 42 (11), 113371. 10.1016/j.celrep.2023.113371 37938972 PMC10955802

[B26] Selman M. Pardo A. (2021). Fibroageing: an ageing pathological feature driven by dysregulated extracellular matrix-cell mechanobiology. Ageing Res. Rev. 70, 101393. 10.1016/j.arr.2021.101393 34139337

[B27] Tacutu R. Thornton D. Johnson E. Budovsky A. Barardo D. Craig T. (2018). Human ageing genomic Resources: new and updated databases. Nucleic Acids Res. 46 (1), D1083–D1090. 10.1093/nar/gkx1042 29121237 PMC5753192

[B28] Tanaka T. Basisty N. Fantoni G. Candia J. Moore A. Z. Biancotto A. (2020). Plasma proteomic biomarker signature of age predicts health and life span. Elife 9, e61073. 10.7554/eLife.61073 33210602 PMC7723412

[B29] Tanaka T. Biancotto A. Moaddel R. Moore A. Z. Gonzalez-Freire M. Aon M. A. (2018). Plasma proteomic signature of age in healthy humans. Aging Cell 17 (5), e12799. 10.1111/acel.12799 29992704 PMC6156492

[B30] Wang S. Li W. Hu L. Cheng J. Yang H. Liu Y. (2020). Naguider: performing and prioritizing missing value imputations for consistent bottom-up proteomic analyses. Nucleic Acids Res. 48 (14), e83. 10.1093/nar/gkaa498 32526036 PMC7641313

[B31] Wederell E. D. de Iongh R. U. (2006). Extracellular matrix and integrin signaling in lens development and cataract. Semin. Cell Dev. Biol. 17 (6), 759–776. 10.1016/j.semcdb.2006.10.006 17134921

[B32] Weinberg J. Gaur M. Swaroop A. Taylor A. (2022). Proteostasis in aging-associated ocular disease. Mol. Asp. Med. 88, 101157. 10.1016/j.mam.2022.101157 PMC9742340 36459837

[B33] Xiao M. F. Xu D. Craig M. T. Pelkey K. A. Chien C. C. Shi Y. (2017). Nptx2 and cognitive dysfunction in Alzheimer's disease. Elife 6, e23798. 10.7554/eLife.23798 28440221 PMC5404919

[B34] Xu Y. Yang W. (2023). Editorial: artificial intelligence applications in chronic ocular diseases. Front. Cell Dev. Biol. 11, 1295850. 10.3389/fcell.2023.1295850 38143924 PMC10740206

[B35] Yan K. Bai B. Ren Y. Cheng B. Zhang X. Zhou H. (2022). The comparable microenvironment shared by colorectal adenoma and carcinoma: an evidence of stromal proteomics. Front. Oncol. 12, 848782. 10.3389/fonc.2022.848782 35433435 PMC9010820

[B36] Yang W. H. Shao Y. Xu Y. W. (2023). Guidelines on clinical research evaluation of artificial intelligence in ophthalmology. Int. J. Ophthalmol. 16 (9), 1361–1372. 10.18240/ijo.2023.09.02 37724285 PMC10475621

