# Supplementary figures and images for: Revealing age-related changes in the intraocular microenvironment and senescence modulators using aqueous humor proteomics and machine learning
Source: Front Cell Dev Biol. 2025 Jul 16;13:1583330. doi: 10.3389/fcell.2025.1583330 (PMC12307501; doi:10.3389/fcell.2025.1583330)

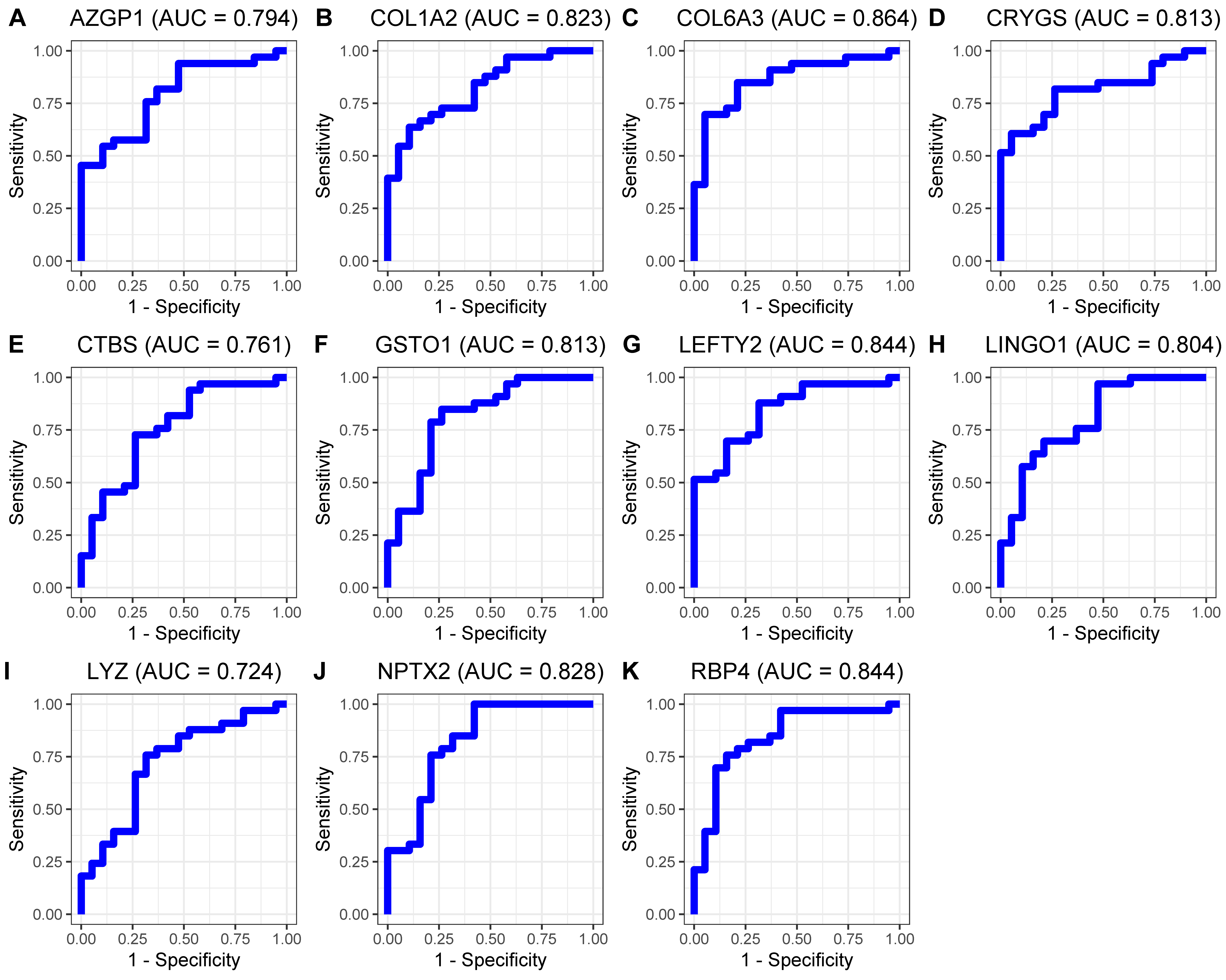

Supplement: Supplementary file 1 [file Image2.jpg]
